# Analysis of risk factors associated with cerebral angiography headache

**DOI:** 10.1055/s-0043-1768157

**Published:** 2023-05-09

**Authors:** Tiago Madeira, Amanda Viguini Tolentino Correa, Gabriela de Paula Abranches, Marcelo Rodrigues Masruha

**Affiliations:** 1 Universidade Federal de São Paulo, Departamento de Neurologia e Neurocirurgia, São Paulo SP, Brazil.; 2 Hospital Santa Rita de Cássia, Departamento de Neurologia e Neurocirurgia, Vitória ES, Brazil.

**Keywords:** Cerebral Angiography, Headache, Migraine Disorders, Headache Disorders, Secondary, Headache Disorders, Angiografia Cerebral, Cefaleia, Transtornos de Enxaqueca, Cefaleias Secundárias, Transtornos da Cefaleia

## Abstract

**Background** Despite previous studies indicating a moderate/high incidence of angiography headache (AH), there is still limited data about the risk factors associated with its occurrence.

**Objective** The present study aimed to assess the associations among demographic, clinical, and technical characteristics of cerebral digital subtraction angiography (DSA) and the occurrence of AH.

**Methods** Cross-sectional analytical observational study with a sample comprised of individuals with a recommendation for elective DSA. Clinical interviews were conducted to assess the occurrence of AH, using a standardized questionnaire.

**Results** Among 114 subjects, the mean age was 52.8 (±13.8) years old, 75.4% (86/114) were women, 29.8% (34/114) had a history of migraines, and 10.5% (12/114) had chronic headaches. The overall frequency of AH was 45.6% (52/114). Of those, 88.4% (46/52) underwent 3D angiography, 7.7% (4/52) underwent aortography, and 1.9% (1/52) underwent both procedures. There was a statistically significant association between AH and previous history of migraine (odds ratio [OR]: 4.9; 95% confidence interval [CI] 1.62–14.7; *p* = 0.005) and 3D angiography (OR 6.62; 95%CI: 2.04–21.5; *p* = 0.002).

**Conclusions** 3D angiography is strongly associated with the occurrence of AH, which has never been reported before. The association between a previous history of migraine and AH confirms the results of previous studies.

## Resumo

**Antecedentes** Apesar de estudos prévios indicarem uma incidência moderada/alta de cefaleia da angiografia (CA), os dados sobre os fatores de risco associados à sua ocorrência ainda são relativamente escassos.

**Objetivo** O presente estudo teve como objetivo avaliar as associações entre as características demográficas, clínicas e técnicas da angiografia cerebral por subtração digital (ACSD) e a ocorrência de CA.

**Métodos** Estudo observacional analítico transversal com uma amostra composta por indivíduos com indicação de ACSD em caráter eletivo. Entrevistas clínicas foram realizadas utilizando um questionário padronizado para acessar a ocorrência de CA.

**Resultados** Entre os 114 indivíduos, a idade média foi de 52,8 (±13,8) anos, 75,4% (86/114) eram mulheres, 29,8% (34/114) tinham histórico de enxaqueca e 10,5% (12/114) tinham cefaleia crônica. A frequência geral de CA foi de 45,6% (52/114). Desses, 88,4% (46/52) foram submetidos à angiografia 3D, 7,7% (4/52), à aortografia e 1,9% (1/52), aos dois procedimentos. Houve associação estatisticamente significativa entre CA e histórico prévio de enxaqueca (odds ratio [OR] 4,9; intervalo de confiança [IC] 95%: 1,62–14,7; *p* = 0,005) e angiografia 3D (OR 6,62; IC95%: 2,04–21,5; *p* = 0,002).

**Conclusões** A angiografia 3D está fortemente associada à ocorrência de CA, o que é inédito na literatura. A associação entre um histórico de enxaqueca e a CA confirma os resultados de estudos anteriores.

## INTRODUCTION

Angiography headache (AH) is defined as a headache that is temporally associated with the performance of cerebral digital subtraction angiography (DSA), which meets the diagnostic criteria established by the third edition of the International Classification of Headache Disorders (ICHD-3), 1 shown in Table 1 . In the literature, reports of its frequency are highly variable (0.3 to 55.6%). 2 3 4 5 6 It is usually underestimated by neuroradiologists, possibly due to its usually benign and transient character. Nonetheless, in our clinical practice, we see that patients with a previous history of AH tend to be resistant to performing new cerebral DSAs. The recommendation to perform subsequent cerebral DSAs is especially frequent in patients who need follow-up after endovascular treatment, as noninvasive imaging methods may not provide as accurate information as cerebral DSAs. In such cases, the expectation to perform a new cerebral DSA may cause patients great suffering and psychological stress, making AH a real clinical issue.

**Table 1:** The ICHD-3 diagnostic criteria for angiography headache

A. Any new headache fulfilling criterion C;
B. Intra-arterial carotid or vertebral angiography has been performed;
C. Evidence of causation demonstrated by at least two of the following: 1. Headache has developed during or within 24 hours of angiography; 2. Headache has resolved within 72 hours after the angiography; 3. Headache has one of the following sets of characteristics: (a) developing during contrast injection and lasting < 1 hour, (b) developing a few hours after the angiography and lasting > 24 hours, (c) occurring in a patient with migraine and having the features of migraine with or without aura.
D. Not better accounted for by another ICHD-3 diagnosis.

Abbreviation: ICHD-3, International Classification of Headache Disorders.

The literature has relatively few investigations of AH and its risk factors. Female sex was associated with AH in the study of Gil-Gouveia et al., 2 Aktan et al., 7 and Demir et al. 6 Among the studies that found an association between AH and previous history of headaches, we name Ramadan et al., 5 Aktan et al., 7 and Demir et al. 6 Regarding demographic factors, a higher education level was associated with AH in the publications of Kwon et al. 4 and Demir et al. 6

Therefore, even though the evidence indicates a moderate/high incidence of AH, there is still limited data about the risk factors associated with its occurrence. The present study aimed to identify the extent to which demographic and clinical factors and the technical characteristics of cerebral DSA are associated with the occurrence of AH.

## METHODS

This is an observational, analytical, cross-sectional study approved by the Federal University of São Paulo ethics committee and all participating hospitals (Research Ethics Committee 93292918.0.0000.5505). Data was acquired from August 2018 to October 2019 in the Department of Interventional Neuroradiology of 5 hospitals in the metropolitan region of Vitória, state of Espírito Santo, Brazil.

The sample comprised patients whose cerebral DSA had been indicated by the principal investigator and patients that other specialists referred to the hospitals participating in the study. Table 2 shows both inclusion and exclusion criteria. The main investigator informed all participants about the nature of the study on the day of their procedure. They were invited to participate upon a signed consent form.

**Table 2:** Inclusion and exclusion criteria of the study

Inclusion criteria	Age between 18 and 80 years old
	Men or women
	Cognition level that allows the analysis of the occurrence of AH
	Consent to the participation in the study, granted by signing an informed consent form
Exclusion criteria	Unstable clinical condition
	Need to use sedative medication to perform the DSA, regardless of type and dose
	Presence of disease and/or neurological sequelae that impair(s) the assessment of the occurrence of AH
	Patients who have presented a change in their clinical and/or neurological condition between the angiography and the post procedure interview with a current condition that makes it impossible to evaluate the occurrence of AH
	Indication for concomitant coronary and/or peripheral vascular angiography
	Patients who have not stopped using metformin for at least 48 hours prior the DSA
	Hemorrhagic or ischemic stroke in the last 90 days

Abbreviations: AH, angiography headache; DSA, digital subtraction angiography.

The principal investigator personally interviewed the participants before the cerebral DSA using forms developed by the authors based on their clinical experience. Information on the patient's sociodemographic profile, comorbidities, and medications in use were collected. Migraine, chronic headache, hypertension, diabetes, overweight, obesity, kidney disease, depression, and anxiety were the comorbidities evaluated in our study. We followed ICHD-3 for diagnostic criteria of migraine and chronic headaches. To measure the other conditions, we considered whether the patient was taking any antihypertensive or antidiabetic medication, had a glucose level ≥ 126mg/dl or a creatinine level ≥ 1.6mg/dl on laboratory tests and had a body mass index (BMI) revealing overweight or obesity on height and weight anthropometry. Depression and anxiety were considered whether the patient had reached the ≥ 9 cutoff point on the translated Hospital Anxiety and Depression Scale (HAD). 8

Immediately after the procedure, the patients were personally interviewed to assess the occurrence of AH. Between the 3 ^rd^ and 7 ^th^ days post-DSA, a new interview was performed by telephone to confirm or exclude the occurrence of AH. All procedures and interviews were conducted exclusively by the main investigator.

All procedures were performed through the femoral route using the iodinated contrast media agent Omnipaque (Iohexol) 300. The number of injected vessels and the complementary performance of aortography and/or 3D angiography varied between subjects, according to the recommendation for the cerebral DSA. All aortographies and 3D angiographies were performed using contrast media pressure injectors. All data related to cerebral DSA was collected immediately after the procedure.

Statistical analyses were performed using IBM SPSS Statistics for Windows, version 24.0 (IBM Corp., Armonk, NY, United States). Descriptive statistics were reported using frequencies and percentages for categorical variables, means and standard deviations (SDs) were reported for normally distributed continuous variables, and medians and interquartile ranges were reported for asymmetrical continuous variables. Categorical and continuous variables were compared using the chi-squared test and the independent Student *t* -test or the Mann–Whitney U test, respectively.

Multivariable binary logistic regression was analyzed to identify the association of the sociodemographic, clinical, and technical risk factors associated with AH. The goodness-of-fit of the regression model was tested using Cox and Snell R-square, Nagelkerke R-square, and Hosmer and Lemeshow tests. The variables considered for inclusion using the stepwise logistic regression approach were based on statistical significance from univariate statistics (p ≤ 0.10), factors that are known to be associated with the presence of AH in previous studies, or the researcheŕs own clinical experience. The covariates included were age, sex, previous history of migraine, depression and anxiety comorbidity (patients who have both diagnosis), hypertension, diabetes, aortography, and 3D angiography. The outcome was described with odds ratios (ORs) and 95% confidence intervals (Cis). There was no missing data. Values of *p* < 0.05 were considered statistically significant.

## RESULTS

A total of 129 subjects were subject to elective DSA over 15 months. Fifteen subjects were excluded (2 for not having discontinued the use of metformin for a period equal to or greater than 48 hours, 6 due to the need for sedation to perform the DSA, and 7 because they had cerebrovascular disease with an ictus date of < 90 days), leaving 114 subjects evaluated in the present study. None of the subjects refused to participate. The overall frequency of AH was 45.6% (52/114).

Table 3 shows the demographic, clinical, and technical data. The mean (SD) age of the whole group was 52.8 (±13.8) years old. The group mainly comprised women (86/114, 75.4%). The main diagnosis observed on angiography was aneurysm (76/114, 66.7%), followed by normal angiography (13/114, 11.4%) and arteriovenous shunt (9/114, 7.9%). Regarding clinical characteristics, 29.8% (34/114) of the participants had a history of migraine, and 10.5% (12/114) had chronic headaches.

**Table 3:** Demographic, clinical, and technical factors reaching statistical significance

Variables		Total	Angiography headache		*p* -value
			No	Yes	
Participants		114 (100.0%)	62 (54.4%)	52 (45.6%)	–
Sex	Female	86 (75.4%)	40 (46.5%)	46 (53.5%)	0.003
	Male	28 (24.6%)	22 (78.6%)	6 (21.4%)	
Migraine	No	80 (70.2%)	52 (65.0%)	28 (35.0%)	< 0.0001
	Yes	34 (29.8%)	10 (29.4%)	24 (70.6%)	
Depression	No	89 (78.1%)	54 (60.7%)	35 (39.3%)	0.011
	Yes	25 (21.9%)	8 (32.0%)	17 (68.0%)	
Anxiety	No	84 (73.7%)	51 (60.7%)	33 (39.3%)	0.023
	Yes	30 (26.3%)	11 (36.7%)	19 (63.3%)	
Hypertension	No	48 (42.1%)	32 (66.7%)	16 (33.3%)	0.025
	Yes	66 (57.9%)	30 (45.5%)	36 (54.5%)	
Mean contrast media volume ± SD (mL)		133.40 ± 40.72	145.38 ± 42.68	124.35 ± 37.13	0.022
		***n *** **= 109**	***n *** **= 58**	***n *** **= 51**	
Pressure injector	No	23 (21.1%)	20 (87.0%)	3 (13.0%)	< 0.0001
	Yes	86 (78.9%)	38 (44.2%)	48 (55.8%)	
3D Angiography	No	30 (27.5%)	24 (80.0%)	6 (20.0%)	0.001
	Yes	84 (73.7%)	38 (45.2%)	46 (54.8%)	

Abbreviation: SD, standard deviation.

In our study, 71.2% (37/52) of the subjects had unilateral headaches, and 28.8% (15/52) had bilateral headaches; for 51.9% (27/52), the headache was stabbing, and 48.1% (25/52) experienced the nonstabbing type of headache. The average maximum pain intensity was 7.8/10.

Regarding the procedure, the mean (SD) contrast media volume used was 133.4ml (±40.7). In our sample, 73.7% (84/114) and 10.5% (12/114) underwent 3D angiography and aortography, respectively. In five patients, both procedures were performed, and they were excluded from the analysis.

A higher proportion of AH than expected was observed in subjects who underwent 3D angiography (46/84; 54.8%; *p* = 0.001), suggesting that the 3D angiography might be associated with AH. It was also interesting to note a significantly smaller mean contrast media volume in the AH group compared with subjects without AH ( *p* = 0.022).

Due to sample size restrictions, the best multivariable regression model was employed with six predictors and is represented in Table 4 . A previous history of migraine (OR: 4.9; 95%CI 1.62–14.7; *p* = 0.005) and 3D angiography (OR: 6.6; 95%CI 2.04–21.5; *p* = 0.002) were significantly associated with the occurrence of AH. Covariate depression and anxiety (OR: 3.74; 95%CI = 0.84–16.5; *p* = 0.082), female sex (OR: 3.02; 95%CI: 0.99–9.2; *p* = 0.052), hypertension (OR: 2.26; 95%CI: 0.89–5.7; *p* = 0.085), and diabetes mellitus (OR: 5.08; 95% CI 0.98–26.2; *p* = 0.052) were not significant in the adjusted model.

**Table 4:** Multivariable binary logistic regression analysis of demographic, clinical, and procedural predictors of angiography headache

Variables	Adjusted OR (95%CI)	*p* -value
Female sex	3.02 (0.99–9.2)	0.052
Hypertension	2.26 (0.89–5.7)	0.085
Diabetes mellitus	5.08 (0.98–26.2)	0.052
Depression and anxiety	3.74 (0.84–16.5)	0.082
Migraine	4.9 (1.62–14.7)	**0.005**
3D Angiography	6.62 (2.04–21.5)	**0.002**

Abbreviations: CI, confidence interval; OD, odds ratio.

Notes: Cox and Snell R-square; 0.32 Nagelkerke R-square; 0.43 Hosmer and Lemeshow test Χ ^2^ = 4.79, df = 8, *p* = 0.779. The numbers in bold are statistically significant.

## DISCUSSION

According to the ICHD-3, the temporal relationship between the supposed triggering factor and the onset of the headache is important in acute secondary headaches. Following this guideline, a patient with a headache starting 24 hours after cerebral DSA could fulfill the criteria for AH (e.g., criteria A, B, C2, C3c, and D). We believe it is inappropriate to establish this diagnosis with a headache starting 24 hours after the procedure. Therefore, we adapted the ICHD-3 diagnostic criteria by excluding subjects whose headaches started > 24 hours after the cerebral DSA.

Significant variation in the frequency of AH can be explained by the heterogeneity of the inclusion criteria used in different studies and the lack of uniformity in their definitions of AH. 3 4 5 6 7

Women predominated in the sample (75.84%), which is consistent with the fact that intracranial aneurysms represented the main recommendation for performing cerebral DSA, as this cerebrovascular disease is more prevalent in women. 9 Despite some studies having found association between AH and female sex, 2 6 7 in the multivariate analysis this factor did not reach statistical significance.

As far as we know, this is the first study that systematically assesses the relationship between psychiatric disorders and AH. The covariate depression and anxiety was not statistically significant in the multivariate analysis. The association between psychiatric disorders and pain could be explained by the fact that these diseases share biological pathways and neurotransmitters. 10

Interestingly, our study found an association between hypertension and the occurrence of AH, which should be analyzed cautiously. Blood pressure was not monitored before, during, or after the procedure. These facts lead to the hypothesis that some cases attributed to AH may represent cases of secondary headaches due to a significant rise in blood pressure (a headache attributed to a hypertensive crisis without hypertensive encephalopathy). In fact, when adjusted for other variables, hypertension was not a significant predictor of AH.

This is the first study showing a strong association between AH and 3D angiography (OR: 6.6). This technological resource allows a highly detailed analysis of intracranial vessels and is essential for analyzing cerebrovascular diseases. The 3D acquisition requires the infusion of contrast media in a greater volume and pressure than those commonly used for conventional acquisitions. Therefore, the observed association may be explained by the fact that the contrast media injected under greater pressure determines an acute mechanical stimulus in the wall of the cervical and cranial arteries, causing pain at the time of the cerebral DSA. This hypothesis had already been raised by Martins et al. when they reported a series of 11 patients who developed headaches during intracranial endovascular procedures. For those authors, the most similar pathogenesis of the pain is the direct stimulation of the arterial wall of the craniocervical arteries. 11 Another evidence that the stimulation of the arterial wall may play a role in the pathophysiology of AH is the study by Aktan et al. 7 These authors assessed 139 subjects who underwent cerebral DSA and 30 control subjects (who underwent peripheral DSA). The frequency of AH was 30.2% in the group subject to cerebral DSAs and 10% in the control group.

The results suggest that a previous history of migraine was associated with a higher chance of AH. This association can be explained by the fact that migraine and AH share similar pathophysiology: the activation of the trigeminovascular system. 2 6 7 12

It is credible to assume that the total contrast media volume used in the cerebral DSA was directly associated with the occurrence of AH. It is interesting to note that there was an inverse relationship between the amount of contrast and the occurrence of AH. In our series, many patients who underwent angiography using a small total amount of contrast usually required 3D acquisition protocols. Thus, we consider that the AH in these patients was caused by the 3D acquisition and not by the use of a small amount of contrast.

The absence of a standardized assessment of the cognitive function of the participants and the fact that the interviews were not blinded represent important limitations of our study. The convenience sample due to budget constraints for the present study was also a significant limitation.

In conclusion, our study found that 3D angiography using a pressure injector is a strong predictor for the occurrence of AH, which has never been reported before. The association between migraine and AH confirms the results of previous studies.

As this is a cross-sectional study, true causality may not be defined yet.

Knowledge of these factors may allow future clinical researchers to assess prophylactic measures in patients at greater risk for developing AH.
